# Burden of* Helicobacter pylori* Infections and Associated Risk Factors among Women of Child Bearing Age in Addis Ababa, Ethiopia

**DOI:** 10.1155/2018/5183713

**Published:** 2018-11-12

**Authors:** Kumera Terfa Kitila, Lemi Mosisa Sori, Daniel Melese Desalegn, Kassu Desta Tullu

**Affiliations:** ^1^Ethiopia Public Health Institute (EPHI), Addis Ababa, Ethiopia; ^2^Addis Ababa University, College of Health Sciences, Department of Medical Laboratory Sciences, Addis Ababa, Ethiopia; ^3^City Government of Addis Ababa Technical and Vocational Education Training Agency, Addis Ababa, Ethiopia

## Abstract

**Background:**

Early detection and treatment of* Helicobacter pylori* (*H. pylori) *infection in women of child bearing ages may reduce the risk of maternal health disorder. This study was conducted to determine the burden of* H. pylori* infections and associated risk factors among women of child bearing ages in Kolfe Keranio Subcity Woreda 9 Health Centers, Addis Ababa, Ethiopia.

**Methods:**

Facility based cross sectional study design was conducted from April to October 2015. The study recruited 195 pregnant and 137 nonpregnant women with age range of 16-40 years. Sociodemographic data of study participants were collected by structured questionnaire. Venous blood was analyzed to determine hemoglobin,* H. pylori* stool antigen test kit was used to assess* H. pylori *infection, and fresh fecal (stool) was used to examine intestinal parasites among study subjects. Data was entered and analyzed using SPSS version 19. Bivariate and multivariate logistic regression model using odds ratio (OR) at 95% confidence interval (CI) were calculated. P-value less than 0.05 was taken as statistically significant.

**Results:**

The overall burden of* H. pylori* infection among study participants was 29% (96/332).* H. pylori* infection was statistically significantly associated with pregnancy status (AOR: 1.825, CI (1.42-2.15), P=0.020), history of hyperemesis gravidarum (AOR=7.028, C.I (2.47-19.99), P=0.018), and low hemoglobin value (AOR=0.177, CI (0.083–0.379), p=0.003). There was no statistically significant association between* H. pylori *infection and sociodemographic characteristics and some expected risk factors like smoking, Khat chewing, alcohol drinking habit, and presence of intestinal parasites.

**Conclusion:**

In this study,* H. pylori *infection was still a public health problem in the study area.* H. pylori *infected women also had high rate of anemia compared to women who had not* H. pylori* infected. Hence clinician and other responsible bodies should give a special attention for women who had been infected with* H. pylori*. Further large case control studies are warranted to understand more the role of* H. pylori*, HG, and other associated risk factors.

## 1. Introduction

### 1.1. Background

Maternal mortality remains a major challenge to health systems worldwide and improving maternal health has been on the global health agenda for many years [1]. Ethiopia has one of the highest rates of maternal mortality in Africa [2].* H. pylori* is an inflammatory gastric bacterial infection especially prevalent in developing countries. It is the most common bacterial infection worldwide, infecting almost half of people in developed countries and 80% of people in developing countries [3]. Study conducted in Ethiopia showed that there are gaps on screening and treatment of infectious diseases including* H. pylori* that could contribute to high maternal morbidity [3].* H. pylori* infection has a role in the pathogenesis of various pregnancy related disorders through different mechanisms, mainly focused on iron deficiency anemia, thrombocytopenia, fetal malformations and fetal growth restriction [4]. Chronic infection with* H. pylori* in pregnant women may also cause hyperemesis gravidarum (HG). This particular complication affects 0.3 to 2.0% of all pregnancies. Previous studies showed that HG has a strong correlation with* H. pylori* infection [5, 6].

Epidemiological studies suggested that anemia presumably due to iron deficiency is correlated to* H. pylori* infection. It is related to a high level risk of low birth weight, prematurity, and perinatal mortality. It is associated with anemia caused by iron deficiency regardless of the presence or absence of peptic ulcer disease.* H. pylori* is a cause of gastrointestinal blood loss, reduction in iron absorption, and increase of iron absorption by the bacteria [7, 8].

Moderate to severe maternal anemia has been associated with an increased risk of poor reproductive outcomes, including low birth weight and preterm birth deliveries [9]. Early detection and treatment of multiple causes of severe anemia may reduce the risk of prenatal and maternal mortality [10].

The burden of* H. pylori* infection among various adults and pediatrics groups in Ethiopia is available and the burden could vary in the range of 80-93%. However, none of them address disease burden among women of child bearing ages [11]. Diagnosis of* H*.* pylori* infection includes invasive techniques (requiring endoscopy) such as rapid Urease test, culture, and histology, and noninvasive methods such as serology, urea breath test (UBT), and stool antigen test. Invasive methods due to ethical issues and UBT because of the use of radioactive materials are prohibited in pregnancy [9, 11]. Serologic testing is the most common noninvasive diagnostic method for* H. pylori *and is relatively inexpensive and convenient;* H. pylori* tested by stool antigen test (HPSA) which is an enzymatic immunoassay to detect bacterial antigen of actual ongoing infection in stool is a reliable noninvasive marker in the primary diagnosis and in the monitoring of posttreatment outcome [9, 11]. Maternal health is complex and it requires different studies to identify the problem in each level of maternity health services. Hence this study aimed to determine the burden of* H. pylori* infection and associated factors among women of childbearing ages in the study area.

## 2. Methods

### 2.1. Study Setting and Context

Facility based cross-sectional study design was conducted among women of childbearing ages from April to October 2015. The study was conducted in Kolfe Keranio subcity, Woreda 9 Health Center, Addis Ababa, Ethiopia. The health centers were delivering service for about 150-200 out patients per day. On average 40-50 pregnant women visited the antenatal care clinics on daily bases. The health facility was well established and trained with different professional staff. The laboratory section was also fairly well equipped with laboratory equipment.

### 2.2. Study Population

Three hundred thirty-two women of child bearing age, who were attended antenatal care clinics and outpatient services in the health centers, were used as study populations.

### 2.3. Inclusion and Exclusion Criteria

All volunteer child bearing ages of pregnant and nonpregnant (16-45 years) women were included. However, women on triple therapy for* H. pylori* infection in the past two weeks of data collection period and presentation with severe medical conditions that necessitated urgent care were excluded from the study.

### 2.4. Sample Size and Sampling Procedures

The sample size was determined using single population proportion formula, considering the assumption that prevalence of* H. pylori* infection was taken as 24.1% previous study conducted in Nigeria [12]. By considering 95% confidence interval (CI) and 5% of marginal level, the total sample size was calculated 332. We factored a 10% nonresponse rate in the sample size calculation. Simple random sampling was used to select the study participants. Each day, before the provision of health education, the names of every pregnant woman attending the antenatal clinic were taken. Fifty percent of the women were then randomly selected until the required sample size was attained. The probability sampling was employed to avoid selection bias.

### 2.5. Data Collection and Laboratory Procedure

A convenience sample of participants was recruited during the study period. For nonpregnant study participants the most common reasons for presentation themselves to outpatients' clinic were generalized medical healthcare seeking and in the same time, for pregnant women study participants the most common reasons for their presentation to the antenatal care clinic in the health center were for their prenatal and antennal care follow-up during pregnancy. Using a standard questionnaire, interviews with adult participants were conducted and information was collected on sociodemographic data and some clinical and behavioral variables.

Stool (fecal) Specimen Collection and Laboratory procedure:* H. pylori* infection was examined by using* H. pylori* stool antigen test kits (CTK Biotech HpSA kit, San Diego, CA 92121 Inc., USA). A random stool (fecal) specimen in a clean, dry receptacle was collected. Then the stool collection device with the specimen's ID number (patient ID sticker) was labeled. The stool collection device was opened by unscrewing the top and used the collection stick to randomly pierce in 2-5 different sites, twisting the collection stick into the fecal specimens to help collection if necessary. All inner grooves of the collection stick were filled with fecal specimen. However, excess fecal specimen on the outside of grooves was scraped off. The collection stick and tighten securely to close the stool collection device was replace. According to the manufacturer leaflet provided with the* H. pylori* test kits the relative sensitivity, relative specificity, and overall agreement were 96.7%, 93.8%, and 94.9%, respectively. Its analytical sensitivity was 100% positive detection rate at 1 ng/mL of pylori lysate antigen in fecal specimens. All reagents were ready to use as supplied. Unopened test devices were properly stored at 2-30°C. It was ensured that the test device is brought to room temperature before opening, when stored at 2-8°C. The test device was stabled through the expiration date printed on the sealed pouch. The kits were not stored in the freezer or exposed to temperatures above 30°C. Lastly the stool collection device vigorously was shacked and ready for* H. pylori* test. An* H. pylori* stool antigen (HPSA) test was performed according to the manufacturer's recommendation. The tests were done with fresh stool samples. The results were evaluated within 10 minutes and tests with any change of color of the test-line were interpreted as positive.

Venous blood samples were collected following standard operating procedures (SOP) by well-trained laboratory personnel and were analyzed using cell dyne methods (Hemocount 30^TS^ hematology analyzer machine, Germany) to determine the hemoglobin level of study subjects and then hemoglobin value <12mg/dl for nonpregnant and <11.0 mg/dl for pregnant women was considered as anemic according to hematology analyzer machine user manual. The manufacturer supplied controls were run every morning to ensure that the analyzer was operating within 2.0 standard deviations. The analyzer automatically sampled blood, processed, analyzed, and printed out the hemoglobin concentration levels.

Intestinal parasites identification was done by direct smear microscopy and formol-ether stool concentration technique. The stool samples were processing based on standard operation procedures described in Monica Cheesbrough [13].

### 2.6. Data Management and Quality Assurances

Before data collection, adequate training was given for data collectors and supervisors. Data collectors were instructed to check the completeness of each data before submission. Quality of data collection process and all laboratory procedures were supervised and monitored by the principal investigator.

### 2.7. Statistical Analysis

The collected data were checked for completeness and consistency and summarized using Microsoft office Excel. The data were coded and analyzed using SPSS version 19.0 (SPSS Inc. Chicago, USA) software. Descriptive statistics was employed for the analysis of demographic data. Association between the burden* H. pylori *infection and risk factors was assessed by Chi-square (X^2^) tests. We used odds ratios as a measure of association, with a 95% confidence interval. Variables with p-values < 0.2 at bivariable analysis and these with biological plausibility with respect to* H. pylori* infection were put into backward stepwise multivariable logistic regression to determine predictors for* H. pylori* infection in study subjects. P-value less than 0.05 was taken as statistically significant.

## 3. Results

### 3.1. Study Subjects Characteristics

From the total 332 study participants that were involved in this study, 195 (58.7%) were pregnant and the remaining were nonpregnant women. The mean (±SD) age of the respondents was 27.3 ± 4.7 years. Majority (82.5%) of the study subjects were married. Sixty-two (18.7%) of study subjects were illiterate. Most (70.2%) of childbearing women lived with a family number of four or more. Among the pregnant women, 44.6% and 28.7% were become pregnant for the first and second time respectively. Sixty percent and 31.7% pregnant women were within a gestational period of 1-12 weeks and 13-24 weeks, respectively. One hundred ninety (57.2%) of study participants had gastrointestinal illness. From the total of pregnant women, 74.4% had history of HG. In this study, stool parasitological investigation showed that 71 (21.4%) of study participants had different intestinal parasites. Regarding the risk factors, 6/332 (1.8%), 1/332 (0.3%), and 2/332 (0.6%) of women had an experience habit of drinking alcohol, cigarette smoking, and Khat chewing, respectively. Majority (81.3%) of them had an experience of drinking tea and coffee. Almost all (99.4%) of them were used tap water for drinking purpose and all of them were wash their hands before meals and after toile. Study participants showed that 71 (21.3%) of them had different intestinal parasites, 190 (57.2%) of them had history of gastrointestinal illness, 61 (18.4%) were anemic, and 145 (74.5%) were pregnant women with hyperemesis gravidarum [Table 1].

### 3.2. *H. pylori* Infections and Risk Factors

The overall burden of* H. pylori* infection among study subjects was 29% (96/332). About 36.0% of women in the age group of 31-35 years were had the highest burden of* H. pylori* infection. Around 43.2% pregnant women with 25-40 weeks gestational age have the highest burden of* H. pylori* infection compared to women within 13-24 weeks, which accounts 29.6%. The burden of* H. pylori *was lowest among women without children, 9/62 (14.5%) compared to women with three or more children; it accounts 9/21 (42.8%). A high increment of* H. pylori *infection was observed among women who had more household members. In this study, another important point showed that 44/61 (72.1%) of the study subjects were* H. pylori* infected and they were anemic [Table 2].

There was no statistically significance association between* H. pylori* infection and sociodemographic characteristics (age group, marital status, educational background, occupational status, and number of people in the household), and some expected behavioral and clinical risk factors like habits of drinking alcohol, cigarette smoking, Khat chewing, drinking tea and coffee, water source for drinking purpose, gestational period (week), gravidity (number of pregnancy), parity (number of children), and presence of intestinal parasites, whereas categorical Chi-square (X^2^) analysis showed that there was statistically significant association between* H. pylori* infection and pregnancy status (X^2^= 68.61, P=0.001), history of hyperemesis gravidarum in pregnant women (X^2^=5.259, P=0.001), history of gastrointestinal illness (X^2^=7.3235, p=0.007), and anemia status (X^2^=68.61, P=0.001) of the study subjects [Table 2].

**Figure 1:**
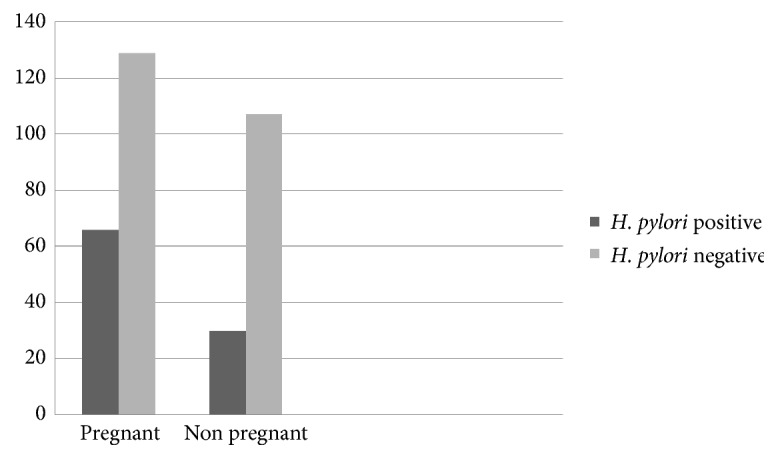
Burden of* H. pylori *infection among women of child bearing ages in a Woreda 9 HC from April to October 2015, Addis Ababa, Ethiopia.

In this study the overall burden of* H. pylori *infection among study subjects was 29%. Burden of* H. pylori *infection among pregnant and nonpregnant women is 21.9% and 33.8%, respectively [Figure 1].

Results of multiple logistic regression analysis showed that pregnant mothers were 1.8 times more likely had* H. pylori *infection than nonpregnant mothers [AOR: 1.825, 95% CI=1.105-3.014, P=0.020]. The pregnant women who had history of hyperemesis gravidarum were seven times more likely had* H. pylori *infection than pregnant mothers without hyperemesis gravidarum [AOR=7.0281, 95% C.I=2.47-19.99, P=0.001]. Women who had gastrointestinal illness were 1.8 times more likely had* H. pylori *infection than who did not infected, even though there was no statistically significant* H. pylori* infection [AOR=1.86, 95% C.I= (0.95-3.667, P=0.060]. Another important point in this study is that also women who had low hemoglobin value were more likely had* H. pylori *infection than who had normal hemoglobin value (AOR=0.177, 95% CI=0.083–0.379, p=0.003) [Table 3].

## 4. Discussion

In this study burden of* H. pylori* infection among pregnant women is lower than study reported 45.2% in Uganda and 52.4% in Belgium, Brussels [14, 15]. However, this study finding is higher than studies conducted in France and Zanzibar that reported a prevalence of 21.5% and 17.5%, respectively [16, 17]. The variation might be due to the difference in study settings, study population, and the laboratory method. In the study of Uganda and Belgium, Brussels, serological antibody test was used as compared to the current study. In the current study burden of* H. pylori *infection among pregnant women was similar to study reported 33.3% in US-Mexico and 24.1% in Nigeria [12, 18].

In the current study,* H. pylori *infection among in the age group of 31–35 years was higher (36.8%) compared to in the age group of 21-25 years (21.4%). This might be due to* H. pylori* acquired during young age but it is asymptomatic, which lead to develop gastritis, peptic ulcers, and gastric carcinoma, usually during late adulthood [11, 16].

The results of this study also showed that* H. pylori* infection was significantly high in the pregnant population with hyperemesis gravidarum accounting 29.6%, and this finding agrees with study conducted in Jeddah, Saudi Arabia, However, in case of Saudi Arabia there was a case control study with hyperemesis gravidarum and without hyperemesis gravidarum [19]. But this study showed higher result than the study done in Izmir, Turkey, which showed that women who were* H. pylori* infected with hyperemesis gravidarum were 22.2% [20]. The possible explanation for this result is that an association of* H. pylori *and hyperemesis gravidarum could be that an increased accumulation of fluid and a displacement of intracellular and extracellular volume occur as a result of increase in steroid hormones, and this condition results in a change of pH which could lead to the manifestation of a latent* H. pylori *infection in the gastrointestinal tract. The increased level of steroid hormones and human chorionic gonadotropins (HCG) during pregnancy also leads to changes in PH and motility of GI tract; this change favors activities of* H. pylori *infection [21].

In this study, 34.7% of* H. pylori* infected study participant women had history of gastrointestinal illness. This finding is relatively lower than study done in Chile, which reported 68.6% of study participant women had* H. pylori *infection among complaining dyspepsia [22]. This indicates that one of the major consequences of* H. pylori* infection is its effect on acid production in the stomach. The bacteria affect the stomach cells that control stomach acid secretion. This can lead to overproduction of hydrochloric acid, paving the way for ulceration, then the acid producing cells themselves are affected, and less acid is secreted, causing low stomach acid. The consequence of low stomach acid is low B12, since it is difficult to assimilate the nutrient from animal protein if you do not have adequate stomach acid, leading to the fact that gastritis or stomach inflammation always accompanies infection [23, 24].

In these study findings, high prevalence of* H. pylori* infection was seen in anemic (72.1%) study women and* H. pylori* infection has been found to be associated with anemia. Our findings are similar to other studies in Baghdad [10], Jeddah, Saudi Arabia [23], Zanzibar [17], and India that showed that women who had been infected with* H. pylori*, indicating that they had high prevalence of anemia [17, 24]. The probable finding of this results could be due to some possible mechanism by which* H. pylori *affects iron metabolism by decreased absorption resulting from chronic gastritis [23], decreased gastric juice ascorbic acid concentration, increased hepcidin production associated with* H. pylori *gastritis, uptake of iron by* H. pylori *for growth, and decreased availability of iron by sequestration of iron in lactoferrin in the gastric mucosa and bacterium host competition for dietary iron supply [14]. Another explanation most commonly offered for this relationship could be also based upon the development of* H. pylori *associated chronic gastritis with resultant achlorhydria and reduced ascorbic acid secretion leading to reduced intestinal iron absorption. Besides an association between anemia [23] and* H. pylori *includes occult blood loss from erosive gastritis [23] and sequestration and utilization of iron by the organism [25].

In this study another point observed is that pregnant women were significantly associated with* H. pylori *infection. These findings agree with that of Cardaropoli and Ehab reports [4, 26]. This probably proposed that pregnant women are among the most vulnerable groups for* H. pylori* infection and a reduction of gastric acid production during early pregnancy as a result of increased accumulation of woman's body fluid, steroid hormone changes, and immunologic tolerance which could lead to the activation of latent* H. pylori* infection, which can exacerbate dyspepsia, nausea, and vomiting symptoms, because of underlying undiagnosed peptic ulcer disease, which in turn may affect maternal gastric absorption. Mild to moderate dyspepsia is commonly associated with nausea and vomiting and complicates about 50% of all pregnancies and it diminishes women's life quality and social functions during early pregnancy [27]. However, in most women, these symptoms resolve by fluid and vitamin supplementation as well as dietary modification.

## 5. Conclusion

In this study* H. pylori *infection was still a problem among women child bearing age in study area.* H. pylori *infection was statistically associated with pregnancy status, low hemoglobin value, and presence of hyperemesis gravidarum in pregnant women. As* H. pylori *infection can lead to serious gastrointestinal problem throughout the individual's life, we would like to emphasize how important it is for* H. pylori* screening and eradication treatment to be conducted in the country. Therefore clinician and other responsible bodies should give a special attention for women who have been infected with* H. pylori*; however further large scale case control studies are warranted among study participants to understand between* H. pylori *infection and related risk factors.

## Figures and Tables

**Table 1:** sociodemographic characteristics and associated risk factors among women of childbearing ages participated in the study in Addis Ababa, Ethiopia, April to October 2015 (n=332).

**Variables**	**Frequency, N**	**Percentage (**%**)**
**Age group**		
16-20	42	12.7
21-25	123	37.0
26-30	94	28.3
31-35	38	11.4
36-40	35	10.6
**Level of education**		
Illiterate	62	18.7
Primary school	119	35.8
Secondary school	73	22.0
College/university	78	23.5
**Marital status**		
Single	51	15.5
Married	274	82.5
Divorced/Widowed	7	2
**Occupational status**		
Government	39	11.7
NGO	19	5.7
Private	128	38.6
House wife	129	38.9
House maid	17	5.1
**Numbers of people in house hold**		
Less than or equal to three	40	12.0
Four	59	17.8
Greater than four	233	70.2
**Numbers of gravidity**		
First pregnancy	87	26.2
Second pregnancy	56	16.9
Three	29	8.7
Greater or equal to four	23	6.9
**Gestational period**		
1-12 weeks	117	35.2
13-24 weeks	62	18.7
Greater than 24 weeks	16	4.8
**Number of child (parity)**		
No child	62	31.7
One child	57	29.3
Two children	55	28.3
Three and above children	21	10.7
**Habit of alcoholism**		
Yes	6	1.8
No	326	98.2
**Habit of smoking**		
Yes	1	0.3
No	331	99.7
**Chewing Khathabits**		
Yes	2	0.6
No	330	99.4
**Water used for drinking purpose**		
Pipe water	330	99.4
Tanker water	2	0.6
**washing hands before meals and after toilets used**		
Yes	332	100.0
**Consumption of tea and coffee**		
Yes	270	81.3
No	62	18.7
**History of Hyperemesis gravidarum**		
Yes	145	74.5
No	50	25.6
**History of gastrointestinal illness**		
Yes	190	57.2
No	142	42.8
**HPSA result**		
Positive	96	29
Negative	236	71
**Presence of intestinal parasite**		
Parasite Seen	71	21.4
Parasite not Seen	261	78.6
**Anemic status**		
Anemic	61	18.4
Not anemic	271	81.6

NGO: nongovernment organization; HPSA: H*. pylori* stool antigen test.

**Table 2:** Univariate analysis showing association of *H. pylori* infection with some associated risk factors among study participated in Woreda 9 HC, Addis Ababa, Ethiopia, April to October 2015 (n=332).

Variables	HPSA positive		X^2^	P-value
	**N**	**n (**%**)**		
**Age group **				
16-20	42	8 (19.1)		
21-25	119	33 (27.7)		
26-30	95	30 (31.6)	3.670	0.453
31-35	50	18 (36.0)		
36-40	26	7 (26.9)		
**Marital status**				
Single	207	17 (8.2)	0.063	0.996
Married	118	77 (65.3)		
Divorced/widowed	7	2 (28.6)		
**Education level**				
Illiterate	36	7 (19.4)		
Primary School	105	30 (28.6)	3.033	0.387
Secondary school	85	23 (27.1)		
Higher education	106	36 (34.0)		
**Occupational status**				
Government	62	22 (35.5)		
NGO	22	4 (18.2)	3.708	0.447
Private	126	35 (27.8)		
house wife	114	34 (29.8)		
house servant	8	1 (12.5)		
**Number of children (parity)**				
No children	62	9 (14.5)		
One	57	22 (38.6)	0.437	0.933
Two	55	18 (32.7)		
Three and above	21	9 (42.8)		
**Gestational week **				
1-12 week	77	26 (33.8)		
13-2 4 week	81	24 (29.6)	7.879	0.069
25-40 week	37	16 (43.2)		
**No. of people in house hold**				
Two	4	2 (50.0)		
Three	76	26 (34.2)	2.479	0.140
Four	138	36 (26.1)		
Greater than four	114	32 (28.1)		
**Gravidity / no. of pregnancy**				
One	91	28 (30.8)		
Two	61	24 (39.3)	8.314	0.140
Greater than three or equal to three	41	14 (34.1)		
**Pregnancy **				
Pregnant	195	66 (33.8)	5.589	0.020*∗*
Non pregnant	137	30 (21.9)		
**Habit of alcohol drinking **				
Yes	6	1 (16.60		
No	325	95 (29.1)	0.446	0.677
**Smoking habit**				
Yes	1	0 (0.0)		
No	331	96 (29.0)	0.468	1.000
**Khat chewing habit**				
Yes	2	0 (0.0)	0.818	1.000
No	330	96 (29.0)		
**Drinking coffee & tea**				
Yes	270	77 (28.5)		0.757
No	62	19 (30.6)	0.111	
**History of gastrointestinal illness**				
Yes	190	66 (34.7)	7.323	0.007*∗*
No	142	30 (21.1)		
**Hyperemesis gravidarum**				
**Yes **	145	61 (29.6)	5.259	0.001*∗*
**No **	50	5 (9.1)		
**water for drinking purpose**				
pipe water	331	96 (29.0)		
Tanker	1	0 (0.0)	0.408	1.000
**Presence of intestinal parasite**				
Negative	261	72 (27.6)	1.049	0.306
positive	71	24 (33.8)		
Anemia status				
Anemic	61	44 (72.1)	68.61	0.001*∗*
Non anemic	271	52 (19.2)		

***Note*.**  *∗*Statistically significant, HPSA: *H. pylori* stool antigen test, and NGO: nongovernmental organization.

**Table 3:** Multivariate logistic regression showing adjusted odds ratio between *H. pylori* infection and associated risk factors among women of childbearing ages participated in the study in selected health centers, Addis Ababa, Ethiopia, April to October 2015 (n=332).

**Variables**	***HPSA *positive**		**COR**	**P-value**	**AOR**	**P-value**
	**N**	**n (**%**)**	** (95**%** CI)**		** (95**%** CI)**	
**Pregnancy status**						
Pregnant	195	66 (33.)	1.570 (1.31, 1.87)	<0.001	1.825 (1.42,2.15)	0.020*∗*
Non pregnant	137	30 (21.9)	1			
**Hyperemesis gravidarum**						
Yes	145	61 (42.1)	6.536 (2.45,17.43)	<0.001	7.028 (2.47,19.99)	0.018*∗*
No	50	5 (10.0)	1			
**Anemia status**						
Anemic	61	33 (54.1)	0.257 (0.144,0.458)	<0.001	0.177 (0.083,0.379)	0.003*∗*
Non anemic	271	63 (23.2)	1			

AOD: adjusted odd ratio, COR: crude odd ratio, CI: confidence interval, and HPSA*: Helicobacter pylori *stool antigen test.

## Data Availability

The data used to support the fndings of this study cannot be shared in a publicly available data repository system, because there is no such a data repository system in the country. However, the data are available from the authors upon request.

## Abbreviations

HG: Hyperemesis gravidarum
HC: Health center
HP: * Helicobacter pylori*
HPSA: * Helicobacter pylori* stool antigen test
HCG: Human chorionic gonadotropins
IDA: Iron deficiency anemia
IQC: Internal Quality Control
SMLS: School of Medical Laboratory Sciences
SOP: Standard operational procedures
WHO: World Health Organization.
